# Regenerative Therapy Modality for Treatment of True Combined Endodontic-Periodontal Lesions: A Randomized Controlled Clinical Trial

**DOI:** 10.3390/ijerph18126220

**Published:** 2021-06-08

**Authors:** Reham AlJasser, Sundus Bukhary, Mohammed AlSarhan, Dalal Alotaibi, Saleh AlOraini, Syed Rashid Habib

**Affiliations:** 1 Department of Periodontics and Community Dentistry, College of Dentistry, King Saud University, Riyadh 11545, Saudi Arabia; malsarhan@ksu.edu.sa (M.A.); dalalotaibi@ksu.edu.sa (D.A.); ssaloraini@ksu.edu.sa (S.A.); 2 Division of Endodontics, Department of Restorative Dental Science, College of Dentistry, King Saud University, Riyadh 11545, Saudi Arabia; sbukhary@ksu.edu.sa; 3 Department of Prosthetic Dental Sciences, College of Dentistry, King Saud University, Riyadh 11545, Saudi Arabia; syhabib@ksu.edu.sa

**Keywords:** endo-perio lesions, periodontal therapy, mineral trioxide, MTA, bone grafting, endodontic therapy

## Abstract

The aim of this in-vivo study was to evaluate/compare the clinical periodontal parameters in patients with true combined endo-perio lesions (EPL), treated with gutta-percha (GP) and mineral trioxide (MTA) as an obturation material alone and with addition of bone grafting in such lesions. 120 Saudi patients (mean age = 41yrs) diagnosed with true combined EPL participated in this study. Group I (control group, *n* = 30) was treated with conventional endodontic treatment using GP for obturation. Group II (*n* = 30) was treated with conventional endodontic treatment using MTA for obturation. Group III (*n* = 30) was treated with conventional endodontic treatment using GP for obturation + grafting procedure to fill the bony defect. Group IV (*n* = 30) was treated with conventional endodontic treatment using MTA for obturation + grafting procedure to fill the bony defect. Clinical parameters (Pocket depth (PD); Clinical attachment loss (CAL); keratinized tissue width (KTW); gingival phenotype (G.Ph.) and Cone Beam Computed Tomography Periapical Index (CBCTPAI)) were recorded and compared at baseline, 3, 6, 12 months’ interval. For the groups III and IV, CBCTPAI showed significant difference (*p* < 0.0001) with the other groups at 6 months and 1-year interval. The group with MTA + bone graft showed 76% and 90% patients with 0 score at 6 months and 1-year follow-up, respectively. Comparison of mean values of PD among study groups at 3 months, 6 months and 1 year showed significant difference at 3 months, whereas the mean PD values of subjects in GP + bone graft showed significantly higher PD values than other 3 groups (*p* = 0.025). Use of GP and MTA for root canal obturation along with periodontal therapy and bone augmentation helps in resolving complex endo-perio lesions. Bone grafting in addition to obturation with MTA was found to be the best treatment strategy in management of EPL cases and is recommended for clinicians who are treating EPL patients.

## 1. Introduction

Preserving the natural dentition is the goal of every dental treatment. Periodontal treatment not only poses the challenge of preserving the natural teeth, but also of restoring the lost periodontal tissue [1]. Lesions of the periodontal ligament and adjacent alveolar bone may originate from infections of the periodontium or tissues of dental pulp. The nature of the pathological communication between the pulp and the periodontium in true combined endodontic-periodontal lesions (EPL) present a dilemma for the clinician in the diagnosis, treatment and prediction of the prognosis of the treatment [2]. The clinical conditions of EPL present with chronic progression in subjects with periodontitis, or in acute form in teeth associated with traumatic injuries or iatrogenic event, with common signs and symptoms of negative or altered pulp response to vitality tests, deep periodontal pockets, bone resorption, purulent exudate, spontaneous pain and tooth mobility [3,4]. EPL have received several classifications based on the primary source of infection [5]. A recent classification for EPL was revised by Herrera et al. in 2017 and proposed based on the present disease status and on the prognosis of the involved tooth [6].

Treatment of EPL should be in an integrated manner with both endodontic therapy and periodontal regenerative procedure to ensure a successful treatment outcome [7]. Evidence has showed that endodontic infection has a negative impact on periodontal healing, therefore, endodontic therapy should be performed first [8,9]. Currently, the root canal system is filled with gutta-percha-based materials (GP) in combination with endodontic sealer in EPL [10]. With advancements in new endodontic materials, mineral trioxide aggregate (MTA) might become a viable alternative treatment option in endodontic treatment of combined EPL. Several clinical studies have showed that the success rate is greater when using MTA as root-end filling in endodontic surgery compared to other materials or with smoothing of the orthograde GP root filling [11,12,13,14]. MTA exhibits superior sealability against bacterial micro leakage, while demonstrating antibacterial and bio-inductive properties that promotes biologic repair and regeneration of periodontal ligaments (PDL) [13,14]. Hence, the use of MTA as an obturation material might ultimately provide long-term benefits that enhance the prognosis and retention of the involved teeth with EPL [15].

True combined EPL has a more compromised prognosis than the other types of endodontic-periodontal diseases and this depends on the severity of periodontal disease and the response to periodontal treatment [5,16]. Kim et al. report that only 17 out of 42 cases of true combined EPL showed complete healing with the reestablishment of the lamina dura at 2 years recall visits [14]. A significant number of diverse treatment approaches were used to regenerate periodontal tissues including guided tissue regeneration (GTR) using barrier membranes, various types or a combination of grafting materials, enamel matrix proteins, and autologous platelet concentrates [17,18]. Cortellini et al. showed that 92% of severely compromised teeth with attachment loss to the apex successfully regenerated after periodontal regeneration at 5-year examination visit [19]. Another clinical study by Oh et al. showed that periodontal regenerative procedures improved the clinical attachment level and radiographic bone level in endodontic-periodontal lesions [20]. Hence, the periodontal regenerative approach should be considered in the treatment of combined EPL.

Another aspect of debate in EPL treatment is whether to graft the bony fenestration defects present or not. This is due to a consistent debate in the literature about the impact of the endodontic treatment on the healing potential of the periodontium [17,18,19,20]. Some authors have explained that this might be related to the size of bony defect and amount of bone destruction. Therefore, when increased periodontal tissue destruction occurs, an additional second procedure of bone grating can be beneficial to improve periodontal status and periodontal healing around the involved tooth, thus improving overall tooth prognosis [21,22]. Several types of bone grafting materials have been used in regenerated such defects ranging from gold standard osteo-inductive grafts (e.g., autografts and allografts) to osteoconductive grafts (e.g., xenografts, alloplasts) [23,24].

The relationship between the pulpal and periodontal tissues has been extensively studied; however, the treatment protocols of managing combined EPL need more investigations. The remarkable potential of MTA to stimulate the biologic mechanisms necessary for repair and retention of involved teeth could contribute as an alternative filling material in combined EPL. Therefore, the aim of this in-vivo clinical study was to evaluate and compare the clinical periodontal parameters in patients with true combined EPL, treated with GP and MTA as an obturation material alone and with the addition of bone graft in such lesions. The null hypothesis was that the evaluation parameters will remain the same in all the patients with EPL treated with the four treatment modalities.

## 2. Materials and Methods

### 2.1. Ethical Approval, Study Population, and Design

This randomized controlled double blinded trial was conducted between October 2017 to November 2018 in accordance with the Helsinki Declaration of 1975, as revised in 2013. The protocol was approved by the Institutional Committee of Research Ethics at the King Saud University, Riyadh, Saudi Arabia (No. E-18-3540) and registered at ClinicalTrials.gov (NCT04274498).

Each participating patient signed an informed consent form after the details of the study had been explained to them. In addition, each patient was informed that they could withdraw from the study at any time without jeopardizing their rights to proceed with dental care at the Dental University Hospital.

The study was performed at the Dental University Hospital of King Saud University, Riyadh Saudi Arabia. 25–55 year-old Saudi patients diagnosed to have an upper anterior non-vital single rooted tooth with true combined EPL were recruited for this study. All subjects were medically fit and had no history of orthodontic, periodontal, or prosthodontic treatment. Exclusion criteria were as follows: (1) uncontrolled systemic disease or condition that alters bone metabolism (i.e., diabetes, osteoporosis, osteopenia, hyperparathyroidism, or Paget’s disease); (2) history of oral cancer, sepsis, or adverse outcomes to oral procedures; (3) long term use of antibiotics (> 2 weeks in the past two months); and (4) use of medications known to modify bone metabolism (i.e., bisphosphonates, corticosteroids). Upper anterior single rooted teeth with true combined EPL confirmed by Cone beam computed tomography radiograph (CBCT) using a limited field of view (FOV) at a 0.200-mm voxel size, 96 kV, and 11.0 mA with an exposure time of 12s using a Planmeca scanner (Planmeca ProMax; Planmeca, Helsinki, Finland) were included in the study. Teeth with fractures, external or internal resorption or associated with pathological cysts were excluded.

The sample size was determined by G Power software where a confidence level was set at 95%, a power level of 80%, and a moderate effect size from the total sample size was calculated to be of 120 teeth (*n* = 120), randomly assigned to each of study’s four groups (*n* = 30/group). For randomization, a locked computer software (Minitab 1.5, Minitab, State College, PA, USA) was used to allocate patients to one of the proposed study’s four groups. Blocked randomization (block size = 3) was performed to maintain equal group size. The allocation result was concealed in a closed envelope and disclosed to endodontist and periodontist only on the day of the appointment.

The details of the study groups and the materials used in the study are presented in Table 1.

### 2.2. Screening and Treatment Visits’ Sequence:

The first dental visit was performed for examination and assigning cases to each study group. The second dental visit included scaling and root plaining around the tooth and RCT performed by a blinded endodontist. The third dental visit was scheduled one week after RCT was completed also with a blinded periodontist to perform periodontal surgery for group III and IV.

### 2.3. Endodontic Treatment

Each patient received local anesthesia of 2% lidocaine with 1:100,000 adrenaline (DENTSPLY Pharmaceutical, York, PA, USA). A rubber dam was placed, and the endodontic access opening was modified using Endo Access bur no. A0164 (Dentsply Maillefer, Ballaigues, Switzerland) and slow speed diamond KGS3203 (Dentsply Maillefer, Ballaigues, Switzerland). Filing was completed to the appropriated determined working length by an electronic apical locator (Root ZX, J. Morita Corp., Osaka, Japan) and peri-apical radiographs. All root canals were instrumented by standardized apical-coronal preparation techniques and were prepared with hand K-files at the established working lengths. Sizes 3, 4, or 5 Reamer T (Pierce Co., Tokyo, Japan) were used to flare the canal orifice of the roots. All apical preparations were enlarged to three sizes greater than the initial size of the file bound at the full working length. Root-canal irrigation with a combination of 2.5% sodium hypochlorite was performed during root canal preparation by a root-canal syringe. After preparation the patency of the apical foramen was confirmed by inserting a #15 K-file 1 mm through the working length. The root canal was irrigated and then dried with paper points (Absorbent paper points; Zippere, West Palm Beach, FL, USA). For GP group; The Obtura II system was prepared according to the manufacturer’s instructions (Obtura II Operator’s Manual 1993). Silver injection needles of 20 and 23 gauge were used for all obturations and a silicone stop was placed 2 to 5 mm from the working length. Root-canal sealer (Canals N, Showa Dental Co., Tokyo, Japan) was placed into the canal using a paper point. At the time of obturation, injection of the thermo plasticized GP was performed twice, separately. First, the needle was inserted in the apical direction until it bound to the canal wall, and the thermo plasticized GP heated to 200°C in the delivery system was injected. The needle was removed after injecting a few millimeters of GP near the tip of the preparation. The softened GP in the apical portion was then vertically condensed to the apex with a hand plugger dipped in alcohol to avoid adherence to the GP. The remaining root-canal space was then back-filled in increments until GP was observed in the cervical aspect of the root.

As for the MTA treatment group; MTA (ProRoot, Dentsply/Tulsa Dental, Tulsa, OK, USA) was mixed according to the manufacturer’s instructions and was compacted in the root canal system. MTA was initially carried to the apical third using an MTA carrier and compacted using wet cotton rolled on a sterile reamer. The entire root canal system was filled with MTA. Furthermore, a squeezed dry cotton pellet was kept in the chamber and the access cavity was sealed with a temporary restorative material (Cavit-G, 3M ESPE, St. Paul, MN, USA). After 48 hours, the temporary restoration was removed and the cavity was restored with a glass ionomer base (Ketac Molar Easymix 3M ESPE) and light cure composite resin (Z 350, 3M ESPE). All RCT treatments were performed by one endodontist (A.Q).

### 2.4. Bone Grafting Surgical Procedure

Local anesthesia of 2% lidocaine with 1:50,000 adrenaline (DENTSPLY Pharmaceutical, York, PA) was applied. Sulcular incision extended to the labial surface of one adjacent tooth on each side and two vertical incisions to enhance visibility and accessibility, full thickness mucoperiosteal flap was reflected on buccal side, and all granulation tissue was removed from buccal bone defect using a surgical curette*. Furthermore, the defect’s dimensions were measured using a periodontal probe*.

Afterwards the defect was packed with DFDBA (cortico-cancellous) (ACE Surgical Supply Co, Brockton, MA, USA) reaching the edges of adjacent bone level, then the grafted site was covered with a resorbable collagen membrane (ACE Surgical Supply Co, MA, USA) that was trimmed to cover the defect extending its edges 2 mm more than the defect dimensions. Flap was repositioned and sutured using a total of six simple interrupted sutures (vicryl restorable (3–0), ACE Surgical Supply Co, MA, USA). Patients returned 7–10 days post-surgery, sutures were removed and wound healing was evaluated. Overall uneventful healing was observed with no adverse events. (e.g., signs of inflammation and pus discharge, infection).

All periodontal surgeries were performed by the same periodontist (R.A).

### 2.5. Follow Up Visits

The follow up dental visit was scheduled 1, 3, 6 and 12 months post full treatment completion with assurance that all included teeth received permanent restoration. Clinical measurements including probing depth (PD) in mm, relative clinical attachment level (r CAL) which is expressed as the distance between cement–enamel junction (CEJ) to the depth at which the probe met resistance in mm, gingival recession which is the distance from the cemento–enamel junction to the depth of the free gingival margin, Keratinized Tissue Width (KTW), and gingival phenotype (G.Ph.) were recorded at baseline, and 3 and 6 and 12 months follow up visits.

### 2.6. Radiographic Evaluation

Radiographic parameters, including Cone Beam Computed Tomography) CBCT) and standardized vertical periapical radiograph, were taken and measured at baseline and 6 months later in order to follow the recommended after treatment protocol for all combined EPL, and The Cone Beam Computed Tomography Periapical Index (CBCTPAI) was used for evaluation of the healing. Success rate was observed and was defined as changes occurring toward level (0) (Intact periapical bone structures) or 1 (diameter of Periapical radiolucency > 0.5–1 mm) at 6 months and 1 year follow up periods [25].

### 2.7. Statistical Analysis

Data analysis was performed using SPSS 26.0 statistical software (IBM Inc., Chicago, IL, USA). Descriptive statistics (mean, standard deviation, frequencies and percentages) were used to describe the study and outcome variables. The one-way analysis of variance followed by Tukey’s multiple comparison test was used to compare the mean values of quantitative variables at each of the time points among the 4 study groups. A non-parametric statistical test (Kruskal-Wallis) was used to compare the mean ranks of categories of CBCTPAI among the 4 study groups at each of the time points. A *p*-value of ≤ 0.05 was used to report the statistical significance of results.

## 3. Results

The comparison of baseline characteristics across the four study groups (GP; MTA; GP+ Bone graft and MTA+ Bone graft) showed, some differences for the variables: gender, tooth type, tooth number and gingival phenotype, but no statistically significant difference in the mean values: Root length (in mm) (*p* = 0.393), Pocket depth (in mm)(*p* = 0.929), Clinical attachment level (in mm)(*p* = 0.983) and Keratinized tissue width (in mm)(*p* = 0.105) (Table 2).

**Figure 1:**
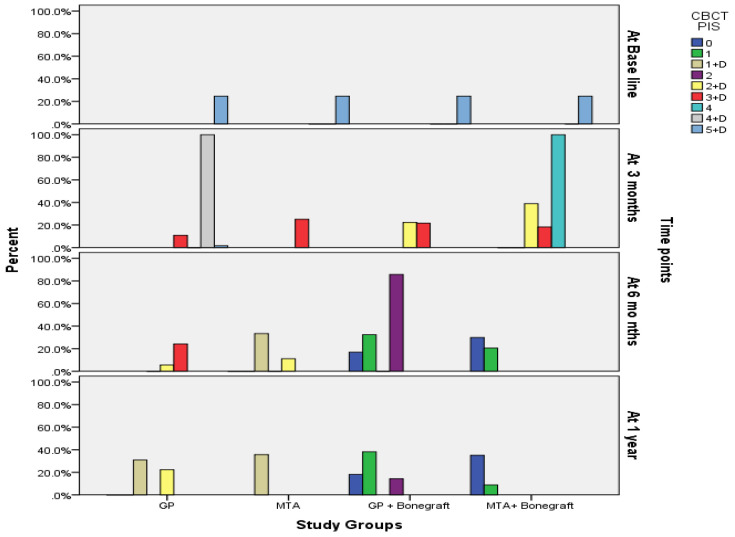
Display of changes in CBCTPAI among the four study groups at each of the four time points of follow up visits.

The comparison and distribution of CBCTPAI among the four study groups at each of the four time points (baseline, 3 months, 6 months and 1 year) showed no statistically significant difference at baseline as all participants across the four groups (GP, MTA, GP+ Bone graft and MTA+ Bone graft) were diagnosed with a periapical lesion and a bone fenestration graded as 5 + D. At 3 months the distribution of CBCTPAI categories showed statistically significant differences. Where in GP group 56.7% of participants had 4 + D and 5 + D in other three groups, no 4 + D and 5 + D grades were diagnosed. At 3 months follow up, all participants (*n* = 30, 100%) in MTA group had 3 + D, whereas (*n* = 26, 86.7%) had 3 + D in GP + bone graft. Furthermore, (*n* = 22, 73.3%) had 3 + D in MTA+ bone graft group when compared with only (*n* = 13, 43.3%) had 3 + D in GP group which is statistically significant (*p* < 0.0001). At 6 months follow up, the CBCTPAI categories were observed as ‘0’ and ‘1’ with 43.3% and 36.7% of participants in GP + bone graft group and 76.7% and 23.3% in MTA + bone graft group. This indicates highly statistically significant difference (*p* < 0.0001 between these groups, with the groups with no bone grafting. Similar statistically significant pattern was observed at 1 year follow up, where higher numbers of participants of two groups that received bone grafting (GP+ bone graft & MTA + bone graft) developed a CBCTPAI categories of ‘0’ and ‘1’ when compared with other opposing groups (*p* < 0.0001). (Table 3) In terms of success rate of treatment, MTA+ bone graft group showed the highest defect fill level as this showed (30) 100% fill of defects at both 6 months and 1 year follow up periods. This was followed by GP + bone graft group which represented by (29) 97% (Figure 1).

The comparison of mean values of PD among the four study groups at each of the three time points (3 months, 6 months and 1 year) showed statistically significant difference at 3 months, where the mean PD values of subjects in GP + bone graft group showed significantly higher PD values than other three groups (*p* = 0.025) (Figure 1). On the other hand, mean PD values at 6 months and 1 year showed only statistically significant difference, whereas GP and MTA groups showed significantly higher PD values when compared to groups received bone grafting (GP + bone graft & MTA + bone graft). The pair wise comparison showed no difference between GP + bone graft and MTA + bone graft, whereas a significant difference between GP and MTA groups was observed showing that GP group had higher PD values both at 6 months and 1 years when compared with MTA group (Table 4).

## 4. Discussion

The present in vivo randomized controlled trial investigated and compared the clinical periodontal parameters in patients with true combined EPL, treated with GP and MTA as a root canal obturation material alone and with the addition of bone grafting in these patients. According to the results of the study variations in the evaluated periodontal parameters were noted/found in the patients with true EPL. Thus, the null hypothesis of no difference in the evaluation parameters for the four test groups was rejected.

Treatment of the adjuvant endodontic and periodontal lesions is challenging for the clinicians as both the endodontic and periodontal treatment must be completed for ensuring successful outcome. Not only the concomitant endodontic and periodontic treatments are complicated, but the clinical procedure is complex, the sequence of procedure must be meticulous and selection of proper materials is critical for the optimal and successful treatment in these EPL cases [26,27,28]. The literature indicates several procedures adopted by different researchers/clinicians over the globe. Most of the researchers agree on and proposes the attainment of endodontic procedure prior to the periodontal therapy, in order to reduce/eliminate the number and presence of bacteria in the root canals, which may become a source of infection and affect the outcome of the periodontal therapy [16,17,18,19,20,21,22,28,29,30,31]. Endodontic treatment eliminates source of infection, and eliminating the entry of pathogenic microorganisms to the periodontium, by blocking the channels of communication between the pulp and periodontal tissues [32]. Consecutive periodontal therapy is essential based on the defect formed. Regeneration, root resection, hemi-section and ozone therapy are also advised as adjunctive procedures for the complete treatment of the EPL lesions in multi rooted teeth [29,30]. Some research studies also reported the usefulness of the bone augmentation during the phase of periodontal therapy for the treatment of EPL as a debate and questions about this aspect are still present [22]. There are data in the literature bout successful treatment in of endo-perio lesions using regenerative procedures such as platelet-rich fibrin and plasma for treatment of combined endo-perio lesions in promoting healing [31].

Prognosis depends on the severity of periodontal disease involvement, efficacy of periodontal repair/regeneration initiated by either of the treatment procedures and patient response to the treatment which may vary from patient to patient. If the majority of bony support has been lost from periodontitis, regardless of predictability of endodontic therapy, the tooth may have a hopeless prognosis.

In the current research two types of materials i.e., GP and MTA were used for the obturation of the root canals along with the periodontal therapy with or without bone augmentation. Conventional root canal obturation with GP along with a root canal sealer has been used as the primary root canal filling material, because of its excellent properties such as handling characteristics and biocompatibility [10]. However, some research studies reported the vulnerability of GP for bacterial/coronal micro leakage [33]. Recently MTA has been evaluated and reported as a viable alternative of GP for root canal obturation. The results of obturation with MTA are promising as it showed improved performance/results in challenging endodontically involved teeth with extensive pathosis that otherwise may not improve or respond to the conventional root canal filling materials and techniques [34]. Few research studies have evaluated the effectiveness of MTA for the treatment of EPL [35,36]. The present study is unique because not only was the effectiveness of MTA was evaluated in true combined EPL, but this was compared with conventional GP as obturation material, as well as investigating the effectiveness of MTA with and without bone augmentation.

The results of the current study revealed a marked improvement in the CBCTPAI of the groups with bone grafting. The data showed that the number of patients with 0 and 1 category with CBCTPAI at 6 months and 1 year follow up were significantly higher (*p* < 0.0001) as compared to the groups with no bone grafting. Among the bone grafting groups the MTA+ bone graft showed even better results with 90% of the patients with ‘0’ CBCTPAI classification. The high success rate with MTA along with bone grafting can be devoted to the excellent physical properties of MTA with regards to hard tissue deposition and to the regenerative technique approach with bone grafting which accelerates the cell differentiation/proliferation/induction and tissue formation. MTA being a bio-ceramic material, with a composition of tricalcium-silicate, tricalcium-aluminate, tricalcium-oxide and silicate-oxide, forms a colloidal gel on hydration that solidifies in about three hours. The calcium-oxide of MTA then forms calcium hydroxide after reacting with the tissue fluids and enhances the hard tissue formation due to its high pH [34]. The bone grafting with allograft further optimizes the tissue remodeling, and accelerates the wound healing and angiogenesis by enhancing the growth factors and proteins [22,23,24]. This was also confirmed by a recent studies evaluating grafting in peri-apical lesions associated with bony defects, as combined GTR techniques (filling material and membranes) obtains a greater success especially in through-and-through lesions of 5mm diameter or more [25] when used with a promising regenerative material such as MTA [26].

The localized immune system may be compromised in patients with true combined EPL [16,28]. It is thus imperative to recognize the beneficial effects that might be achieved with bone grafting procedures in such clinical cases. Conventional cleaning of the pockets with flap surgeries without regenerative procedures may not help in treating the patient successfully. Endo-perio lesions are challenging problems faced by clinicians, and, although they are relatively rare in clinical practice, they can severely compromise the tooth prognosis. The key to successful treatment of the true combined EPL is to make a correct diagnosis, a well-planned and sequenced treatment with multidisciplinary approach, selection of the appropriate materials and the optimal execution of the treatment plan with recommended techniques [9,21,22].

Further randomized clinical trials with large samples sizes are essential to prove the efficacy of use of e bone grafting in such defects and to determine the efficiency of MTA use as well. This is recommended and will generate high-quality evidence with regards to the treatment options for patients with true combined EPL. However, due to the relatively uncommon EPL cases and the variety of clinical parameters associated (for which the standardization is a big challenge), there are some limitations to these type of research studies. In addition, the individualized variations in the response of patients to the different treatment strategies makes the research/job further complicated. It is also recommended for clinicians who are treating EPL cases to review their clinical cases periodically. The reporting of these cases may be valuable for other clinicians to define/refine their treatment plans and anticipate particular clinical outcomes.

## 5. Conclusions

A true combined endo-perio lesion may present with multiple pathogenesis ranging from simple to complex, rendering the outcome of the treatment unpredictable. The lesion from the root canal infection and periodontium must be treated with endodontic and periodontal treatment, respectively. Use of GP and MTA for root canal obturation along with periodontal therapy and bone augmentation helps in resolving the complex endo-perio lesions. Bone grafting in addition to obturation with MTA was found to be the best treatment strategy in management of EPL cases and is recommended for clinicians who are treating EPL patients. During the treatment planning for the EPL cases, an interdisciplinary approach is vital for the success/favorable outcomes of the treatment.

## Figures and Tables

**Table 1:** Details of the tested groups and materials used in the research study.

S. No.	Group (*n* = 120)	Treatment	Obturation/Graft Material	Trade Name	Lot Number
1.	Group-I (*n* = 30)	Conventional non-surgical RCT performing standard methodology	Gutta Percha	Obtura Gutta Percha Bar PK/100 Bar	FD-MT057
2.	Group-II (*n* = 30)	Conventional non-surgical RCT performing standard methodology	Mineral trioxide aggregate	ProRoot MTA, White, 10 × 0.5 g	MP-A040500000400
3.	Group-III (*n* = 30)	Conventional non-surgical RCT performing standard methodology + Bone grafting	Gutta Percha	Obtura Gutta Percha Bar PK/100 Bar	FD-MT057
			Bone graft	AlloOss. Natural Blend Cortico/Cancellous Particulate, 500–1000 mic.	SKU: 01-108-201
4.	Group-IV (*n* = 30)	Conventional non-surgical RCT performing standard methodology + Bone grafting	Mineral trioxide aggregate	ProRoot MTA, White, 10 × 0.5 g	MP-A040500000400
			Bone graft	AlloOss. Natural Blend Cortico/Cancellous Particulate, 500–1000 mic.	SKU: 01-108-201

**Table 2:** Baseline characteristics of study subjects across the 4 study groups.

Characteristics	Study Groups			
	GP	MTA	GP + Bone Graft	MTA + Bone Graft
**Gender**				
Male	18 (60)	9 (30)	8 (26.7)	18 (60)
Female	40 (40)	21 (70)	22 (73.3)	12 (40)
**Tooth type**				
Canine	6 (20)	5 (16.7)	9 (30)	5 (16.7)
Central incisor	7 (23.3)	5 (16.7)	1 (3.3)	6 (20)
Lateral incisor	16 (53.4)	18 (60)	16 (53.4)	17 (56.6)
Premolar	1 (3.3)	2 (6.6)	4 (13.3)	2 (6.7)
**Tooth number**				
11	3 (10)	5 (16.7)	0	2 (6.7)
12	5 (16.7)	9 (30)	6 (20)	5 (16.7)
13	3 (10)	3 (10)	4 (13.3)	2 (6.7)
21	4 (13.3)	0	0	4 (13.3)
22	11 (36.7)	9 (30)	12 (40)	13 (43.3)
23	3 (10)	2 (6.7)	5 (16.7)	3 (10)
24	193.3	2 (6.7)	3 (10)	1 (3.3)
**Gingival phenotype**				
Thick	1 (3.3)	3 (10)	1 (3.3)	0
Flat and thick	18 (60)	13 (43.3)	17 (56.7)	18 (60)
Scalloped and thin	11 (36.7)	14 (46.7)	12 (40)	12 (40)
**Root length (mm)**				
	13.85 (1.48)	13.20 (1.79)	13.77 (1.72)	13.40 (1.69)
**Pocket depth PD (mm)**				
	4.93 (0.91)	5.03 (0.72)	4.90 (0.84)	4.97 (0.72)
**Clinical attachment level CAL (mm)**				
	3.97 (0.93)	4.03 (0.72)	3.97 (0.81)	3.97 (0.67)
**Keratinized tissue width (mm)**				
	2.63 (1.13)	2.10 (0.84)	2.70 (1.18)	2.70 (1.21)

**Table 3:** Comparison of distribution of CBCTPAI among the 4 study groups at each of the 4 time points of follow up visits.

Outcome Variable & Time Point	Study Groups				*p*-Value *
	GPA	MTA	GPA + Bone Graft	MTA + Bone Graft	
**At Baseline**					
5 + D	30 (100)	30 (100)	30 (100)	30 (100)	
**At 3 months**					
2 + D	0	0	4 (13.3)	7 (23.3)	<0.0001 *
3 + D	13 (43.3)	30 (100)	26 (86.7)	22 (73.3)	
4	0	0	0	1 (3.3)	
4 + D	15 (50)	0	0	0	
5 + D	2 (6.7)	0	0	0	
**At 6 months**					
0	0	0	13 (43.3)	23 (76.7)	<0.0001 *
1	0	0	11 (36.7)	7 (23.3)	
1 + D	0	28 (93.3)	0	0	
2	0	0	6 (20)	0	
2 + D	1 (3.3)	2 (6.7)	0	0	
3 + D	29 (96.7)	0	0	0	
**At 1 year**					
0	0	0	14 (50)	27 (90)	<0.0001 *
1	0	0	13 (46.4)	3 (10)	
1 + D	26 (86.7)	30 (100)	0	0	
2	0	0	1(3.6)	0	
2 + D	4 (13.3)	0	0	0	

* *p* value.

**Table 4:** Comparison of mean PD values among the 4 study groups at each of the 4 time points of follow up visits.

Time Point	Study Groups				F-Value	*p*-Value
	GP	MTA	GP + Bone Graft	MTA + Bone Graft		
At 3 months	3.0 (0.00)	3.0 (0.00)	3.10 (0.30)	3.0 (0.00)	3.22	0.025
At 6 months	4.37 (0.76)	3.83 (0.75)	3.27 (0.45)	3.00 (0.00)	33.08	<0.0001 *
At 1 year	4.80 (0.89)	3.83 (0.75)	3.30 (0.59)	3.00 (0.00)	44.11	<0.0001 *

* *p* value.

## Data Availability

Data is available on request from corresponding author.
